# *Citrus aurantium* L. Active Constituents, Biological Effects and Extraction Methods. An Updated Review

**DOI:** 10.3390/molecules26195832

**Published:** 2021-09-26

**Authors:** Sawssan Maksoud, Roula M. Abdel-Massih, Hiba N. Rajha, Nicolas Louka, Farid Chemat, Francisco J. Barba, Espérance Debs

**Affiliations:** 1 Department of Biology, Faculty of Arts and Sciences, University of Balamand, P.O. Box 100, Tripoli 1300, Lebanon; sawssan.maksoud@std.balamand.edu.lb (S.M.); roula.abdelmassih@balamand.edu.lb (R.M.A.-M.); esperance.debs@balamand.edu.lb (E.D.); 2 Ecole Supérieure d’Ingénieurs de Beyrouth (ESIB), Saint-Joseph University, CST Mkalles Mar Roukos, P.O. Box 11-514, Riad El Solh, Beirut 1107 2050, Lebanon; hiba.rajha@usj.edu.lb; 3 Centre d’Analyses et de Recherche, Unité de Recherche Technologies et Valorisation Agro-alimentaire, Faculté des Sciences, Saint-Joseph University, P.O. Box 17-5208, Riad El Solh, Beirut 1104 2020, Lebanon; nicolas.louka@usj.edu.lb; 4 GREEN Extraction Team, INRA, UMR408, Avignon University, F-84000 Avignon, France; farid.chemat@univ-avignon.fr; 5 Nutrition and Food Science Area, Preventive Medicine and Public Health, Food Science, Toxicology and Forensic Medicine Department, Faculty of Pharmacy, Universitat de València, Avenida Vicent Andrés Estellés, s/n, 46100 Burjassot, València, Spain

**Keywords:** *C. aurantium*, active constituents, medicinal uses, biological effects, extraction methods

## Abstract

*Citrus* genus is a prominent staple crop globally. Long-term breeding and much hybridization engendered a myriad of species, each characterized by a specific metabolism generating different secondary metabolites. *Citrus aurantium* L., commonly recognized as sour or bitter orange, can exceptionally be distinguished from other *Citrus* species by unique characteristics. It is a fruit with distinctive flavor, rich in nutrients and phytochemicals which possess different health benefits. This paper presents an overview of the most recent studies done on the matter. It intends to provide an in-depth understanding of the biological activities and medicinal uses of active constituents existing in *C. aurantium*. Every plant part is first discussed separately with regards to its content in active constituents. All extraction methods, their concepts and yields, used to recover these valuable molecules from their original plant matrix are thoroughly reported.

## 1. Introduction

*Citrus*, genus of the family Rutaceae, includes various species of diverse sizes and forms, commonly known as lemons, limes, oranges, mandarins, citrons and grapefruits [1,2]. They are one of the central horticultural crops with universal agricultural production with ≈100 million tons per year [3]. Previously, *Citrus* plants were associated with herbal medicine in many Asian countries such as Japan, China, and Korea. They are in recent years commercialized for their fruits and juice, or used as additives in several industries [4]. Other than being rich in vitamin C and vitamins B, *Citrus* fruits contain minerals, macronutrients such as carbohydrates, dietary fibers, crude proteins, lipids, and phenolic compounds with important health-promoting properties [5,6]. On the other hand, essential oils obtained from *Citrus* species are extensively used in food and beverages, perfumes, pharmaceutical, and cosmetic industries [7].

*Citrus aurantium* L., also known as sour orange, bitter orange, Seville orange, or bigarade, is an evergreen tree that can grow up to 5 meters tall. Renowned for its scented white flowers, it is believed to have originated in eastern Africa and Syria and was cultivated in the United States, Spain, and Italy [8]. A large number of studies were carried out on the bioactivity of *C. aurantium* compounds. The focus of this review article is to compile and document the recent studies performed over the last decade on the bioactive molecules existing in *C. aurantium* plant, unraveling their biological effects and potential medicinal virtues. Furthermore, methods used for their extraction from the original plant parts, are comprehensively overviewed.

## 2. *C. aurantium* Active Constituents

**Figure 1:**
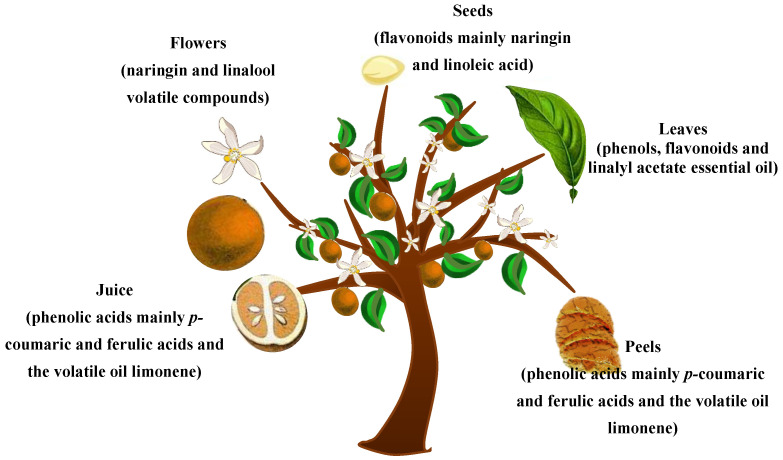
*C. aurantium* (bigarade) tree with the studied plant parts and the most abundant active constituents.

Plant secondary metabolites, or phytochemicals, have well-established properties that are of pivotal importance to human health, comprising, among others, anti-cancer, antiproliferative, hypolipidemic, and cardio-protective activities [8]. Their potent antioxidant activity is evidently linked to their natural ability to hunt free radicals and disrupt radical chains. The *C. aurantium* plant is teeming with phytochemicals. Flavonoids, the major bioactive compounds contained in *C. aurantium*, are grouped into flavanones, flavones and flavonols. Limonoids, such as limonin and nomilin, and alkaloids, such as *p*-synephrine, are also encountered [9]. In the following, and as shown in Figure 1, *C. aurantium* parts including juice, flowers, seeds, leaves and peels are methodically discussed regarding their content in bioactive molecules. It is noteworthy to mention that the chemical composition or the percentage of biomolecules is obviously affected by the geographical area, growing season, and the period of harvest.

### 2.1. C. aurantium Juice

*C. aurantium* juice is mainly used in salad dressings as an alternative to lemon juice, providing its typical flavor [10]. *C. aurantium* juice was reported to contain 86% of phenolic acids out of total phenolic compounds [11]. In a study on Tunisian bitter orange, Jabri Karoui and Marzouk (2013) demonstrated that aroma compounds consisted mainly of monoterpene hydrocarbons including the volatile limonene (92%), followed by α-phellandrene (2%), and α-thujene (1%). Oxygenated sesquiterpenes were found in the juice as well with caryophyllene oxide as the main component (1.4%). Regarding phenolic compounds, phenolic acids represent alone 71% with *p*-coumaric (18%) and ferulic acids (19%) as the most common ones, followed by flavonoids reaching 23% with rutin being the principal one [12]. According to a more recent study undertaken on some *Citrus* varieties, the total phenol content of *C. aurantium* juice was 295 ± 4 mg GAE/g (gallic acid equivalent/g of fresh juice) and the flavonoid content was 26 mg Eq Q/g (quercetin equivalent per g of fresh juice) [13]. This study demonstrated that juice extracts of *C. aurantium* and *C. maxima* varieties exhibited the highest levels of total phenols and antioxidant activities when compared to *C. clementina*, *C. limon*, and *C. sinensis*.

### 2.2. C. aurantium Flowers

*C. aurantium* flowers are widely used in the Mediterranean region as a food flavoring agent, and in several beverages and pastries. They are also used in medical products for their anti-depressant, anti-infectious, and sedative properties, and in skin care products [14]. Bitter orange flowers contain several products comprising essential oils, the hydrosol, and the absolute. The hydrosols are the coproducts of the hydrodistillation or the steam distillation of aromatic plants. They are valuable essential oils that are less abundantly present [15]. Absolute is a mixture obtained from the flowers of aromatic plants using ethyl alcohol as an extraction solvent (after precipitating the waxes) [16].

The total phenolic content (TPC) and the total flavonoid content (TFC) in *C. aurantium* bloom extract represented 4.5 mg GAE/g DW (dry weight) and 4 mg rutin equivalent/g DW respectively [17]. Using RP-HPLC, phenolic and flavonoid contents were identified as gallic acid, caffeic acid, syringic acid, rutin, pyrogallol, naringin and quercetin. A more recent paper showed that the highest TPC and TFC recorded were 81 ± 3 mg GAE/g extract and 20 ± 3 mg QE/g extract respectively in *C. aurantium* flowers’ ethanolic extract, as compared to 1.5 mg GAE/g TPC and 0.4 mg QE/g TFC of the essential oil fraction [16]. Using GC-MS analysis, the same authors identified seventy-seven compounds in the essential oil of *C. aurantium* flowers, with the most frequent chemical classes being oxygenated monoterpenes, aliphatic hydrocarbons, monoterpene hydrocarbons, and esters [16]. Similarly, the most frequent detected compounds in *C. aurantium* petals powder were: D-glucuronic acid, D-limonene, octadecenoic acid, daphnetin, hexadecanoic acid, linalool, pyrrolidinone, and phthalic acid [18]. The latter compounds were listed in descending order: 10%, 5.5%, 4%, 3.7%, 2%, 2%, 1.2%, and 1% respectively.

### 2.3. C. aurantium Seeds

*Citrus* seeds are known to contain bioactive constituents including limonoids, carotenoids, and phenolic compounds. They were used in Persian medicine as analgesic, anti-irritant properties, and as antidotes against poisons and toxins [19]. As commonly reported in the literature, the most abundant flavonoids in *C. aurantium* seeds are hesperidin, neohesperidin, naringin, and narirutin. These compounds are of great importance to human health due to their anti-inflammatory, anti-cancer, anti-oxidative, and cardiovascular protective properties [20].

Flavonoids were reported to be the major components (56%) in *C. aurantium* seeds at the mature stage, whereas phenolic acids were found at more moderate levels (22%) [21]. Flavonoids detected were epigallocatechin, naringin, hesperidin, neohesperidin, naphtorecinol, apigenin, quercetin, resorcinol, catechin, rutin, and kaempherol. Phenolic acids included gallic acid, vanillic acid, syringic acid, rosmarinic acid, *p*-coumaric acid, and trans-2-hydroxycinnamic acid. A more recent study investigating the concentration of phenolic compounds present in different *Citrus* seeds corroborates these findings [22]. Phenolic acids found in bitter orange seeds belong to the hydroxybenzoic acids (vanillic acid, 3.3 µg/g DW) and hydroxycinnamic acids (caffeic acid, 5 µg/g DW; trans-ferulic acid, 3 µg/g DW; and *p*-coumaric acid, 15 µg/g DW) families [22]. TPC yield was nearly 2.5 mg GAE/g DW. In regard to limonin in *C. aurantium* seeds, its yield varied between 0.5 and 0.6 mg/g DW when using Na-Sal and Na-CuS as hydrotropes for extraction, respectively [23].

Based on a different perspective, Hamedi and coworkers (2019) studied the phytosterols and fatty acid profiles of *C. aurantium* seed oil. They identified diverse phytosterols including free campesterol (4 mg/g), esterified and free β-sitosterol (2 mg/g and 33 mg/g respectively), and free stigmasterol (10 mg/g). Interestingly, the major fatty acids identified in the seed oil were linoleic acid (50%) and oleic acid (30%) known as omega-6 and omega-9, in addition to other fatty acids (cerotic acid, stearic acid, arachidic acid, palmitic acid, and palmitoleic acid) [19].

### 2.4. C. aurantium Leaves

*Citrus* leaves are also a significant source of bioactive constituents including flavonoids, ascorbic acid, and phenolic constituents that are recognized as natural antioxidants [24]. *C. aurantium* leaves can be used in pharmaceutical industries since they can be integrated in drug formulations [25].

Many studies were done on *C. aurantium* leaves; they are known to contain various essential oils including mainly limonene, linalool, α-terpineol, and linalyl acetate [26,27]. Phytochemical analysis of *C. aurantium* leaves reflected the presence of several compounds including flavanoids, phytosterols, carbohydrates, saponins, volatile oil, tannins, terpenoids, and proteins [28]. In the same study, 35 compounds were identified after reading the GC-MS profile of *C. aurantium* essential oil obtained through hydrodistillation. The major essential oils identified were eucalyptol (43%), sabinene (17%), β-linalool (15%), α-terpineol (8%), α-pinene (1.3%), β-myrcene (1.2%), 4-terpineol (1.1%), β-pinene (1%), D-limonene (1%), and O-cymene (1%) [28].

TPC and TFC in seven species of *Citrus* leaves (*C. clementina*, *C. aurantifolia*, *C. limon*, *C. navel*, *C. hamlin*, *C. aurantium*, and *C. grandis*) were studied. The TPC of *C. aurantium* leaves in aqueous extracts was 70 ± 2 mg GAE/g DW, but it was only 8 mg GAE/g DW in the methanolic extract. TFC in *C. aurantium* leaves was 12 ± 2 mg QE/g DW in aqueous extract compared to 5 mg QE/g DW in the methanolic extract [24], confirming that aqueous extraction was more efficient in extracting phenolics and flavonoids. In the same way, a study performed in Algeria on the peels and leaves of seven varieties of oranges showed that *C. aurantium* L. cv. *Bigarade* leaves had the highest level of TPC [29]. The authors listed the phenolic compounds of *C. aurantium* leaves as follows: total phenols (44 GAE/g DW), flavonoids (3 mg QE/g DW), flavonols (1.5 mg QE/g DW), proantho-cyanidins (4.5 mg CE/g DW), hydrolyzable tannins (33 ± 2 TAE/g DW), polymerized phenols (8 mg TAE/g DW), and soluble phenols (3 mg GAE/g DW). Furthermore, Haraoui and coworkers (2020) examined the TPC in leaf extracts and juice of ten varieties of *Citrus* thriving in Algeria. *C. aurantium* leaves exhibited the highest level of total phenols (107 ± 2 mg GAE/g DW) as compared to other *Citrus* species, and one of the highest flavonoid’s content (14 mg Eq Q/g DW) [13]. The latter two studies emphasized the richness of *C. aurantium* in phenolic and flavonoid compounds as compared to other *Citrus* species.

### 2.5. C. aurantium Peels

At an industrial scale, the juice yield from *Citrus* is approximately half the fruit weight, while the residual part mass is mostly made of peels (40–50%). Thousands of tons of *Citrus* peels are produced yearly as waste from processing industries. They can cause serious problems for disposal and can greatly pollute the environment since only a small amount is utilized and the remaining bulk is burned [30]. Nevertheless, these peels contain a multitude of volatile oils such as sesquiterpenes, monoterpenes and their derivatives, and many other constituents including flavones and alkaloids such as octopamine, synephrine, N-methylthyramine, and carotenoids [31,32]. They are considered as safe products commonly used in various industries: in cosmetics, perfumes, body care products, and soap industries (due to their marketable fragrance) and in foods, beverages, and ice cream industries as flavoring and acidifying agents [33,34,35,36].

*C. aurantium* has a thick peel that is richer in pectin than the sweet orange peel [25], and that contains higher amounts of essential oils when compared with other *Citrus* species [37]. The essential oil content in *C. aurantium* peels ranged between 0.1 and 1.7% [36] with limonene being the most abundant volatile component [38,39]. Details of compounds found in Italian *C. aurantium* peel were provided as follows: monoterpene hydrocarbons representing 72.5% while oxygenated monoterpenes representing 7% with the major component limonene (66%) [40]. Recently, a slightly different chemical composition of the essential oils of *C. aurantium* peel was reported. The major volatile components identified were monoterpene hydrocarbons (51%) and oxygenated monoterpenes (46%)—mainly: limonene (49%), linalool (32%), linalyl acetate (12%), myrcene (1.2%), geranial (1%), neral (0.5%), β-pinene (0.5%), γ-terpinene (0.4%), sabinene (0.3%), geranyl acetate (0.2%), and β-caryophyllene (0.1%) [41]. Additionally, Jabri Karoui and Marzouk (2013) studied the bioactive contents of Tunisian *C. aurantium* peel. They investigated the aroma compounds using gas chromatography (GC), and gas chromatography mass spectrometry (GC-MS), and the phenolic compounds using reversed-phase high-performance liquid chromatography. The major volatile compound found in *C. aurantium* peel was limonene (90%) and the main phenolic compounds were phenolic acids (74%) followed by flavonoids (23%). The most common phenolic compounds found in the peel of *C. aurantium* were *p*-coumaric (25%) and ferulic acids (24%) [12]. Finally, pectin yield from *C. aurantium* peel was around 28% (with TPC 40 ± 3 mg GAE/g of pectin), consisting of 65% of galacturonic acid [42]. Using a different extraction method, the same team previously showed that pectin yield in *C. aurantium* peel was 29%, containing 71% of galacturonic acid [43].

## 3. Biological Effects of *C. aurantium* Active Constituents

### 3.1. Antioxidant Effect

*C. aurantium* is an evident great source of natural antioxidants. Its leaves were used in many folk traditions for medicinal purposes: for insomnia, stomach aches, and heart palpitations by European Basque people, and as laxatives, relaxing agents for insomnia, and a sedative for tired nerves in South America and Mexico [25]. *C. aurantium* leaf extracts displayed the highest antiradical activity (92.5%) using the stable radical 1,1-diphenyl 1-2-picrylhydrazyl (DPPH) [29]. It also had the highest capacity to slow the oxidation level of β-carotene and linoleic acid (77%) when compared to other orange varieties using the β-carotene bleaching assay [29]. In another study, the antioxidant activities of *C. aurantium* aqueous leaf extract were IC50 72 ± 1 µg/mL, 728 ± 9 µM TE/g DW, and 19 ± 2 mg BHAE/g DW as a result of DPPH scavenging activity, scavenger ABTS radical activity, and FRAP assay respectively [24]. However, the total antioxidant activities were lower in methanolic extract with IC50 68 ± 4 µg/mL using DPPH assay, 354 ± 4 µM TE/g DW using ABTS assay, and 13 mg BHAE/g DW using FRAP assay [24]. According to the same study, the ferric reducing antioxidant activity of *C. aurantium* methanolic extract was among the highest when compared to *C. clementina*, *C. aurantifolia*, *C. hamlin*, *C. limon*, *C. grandis*, and *C. navel*.

Using the phosphomolybdenum method, the total antioxidant activity of *C. aurantium* peel extracts was higher than that of the pulp extracts [44]. The authors attributed this effect to the chemical composition of the peel that is rich in phenolic acids and their derivatives. In the peel extracts, higher antioxidant activity was obtained in methanol (1618 μmol/g) than in water (1522 μmol/g) peel extract. In contrast, the pulp extracts exhibited the highest antioxidant activity in water (637 μmol/g), followed by methanol (467 μmol/g) pulp extract [44]. Moreover, the antioxidant activity of *C. aurantium* fruit extracts was found to be 0.8 mg/mL, 0.5 mg/mL, 4 mg/mL, and 842 ± 20 mM TE/g dry fruit extract using DPPH IC50, ABTS IC50, FRAP (ferric reducing antioxidant power) IC50 and ORAC (oxygen radical absorbance capacity) IC50 assays, respectively [45].

### 3.2. Antimicrobial Effect

*Citrus* fruit extracts can act as natural antimicrobials, offering the possibility to be used in several food applications [46]. Water, ethanol, and chloroform extracts of *C. aurantium* leaves exhibited antibacterial activity against gram-positive (*Staphylococcus aureus* and *Bacillus subtilis*) and gram-negative bacteria (*Escherichia coli* and *Klebsiella pneumonia*) [47]. The effect of *C. aurantium* juice on two bacterial isolates: *Listeria monocytogenes* and *Salmonella* Typhimurium, was investigated. Results showed that tested microorganisms were able to survive in pH-neutralized juice for only two days; however, they could not grow after seven days of incubation [33]. The antimicrobial activity of the juice and leaf extracts of ten varieties of *Citrus* plants grown in Algeria was explored using the agar well diffusion method [13]. *C. aurantium* juice extracts exhibited higher antibacterial activity against all tested gram-positive (*S. aureus*, *B. subtilis*, *M. luteus*, *E. faecalis* and *S. epidermidis*) and gram-negative bacteria (*Klebsiella*, *P. aeruginosa* and *E. coli*) than leaf extracts. *C. aurantium* and *C. limon* showed the highest antimicrobial activity, followed by grapefruit and mandarin. The authors attributed the difference in the degree of sensitivity of microorganisms to their intrinsic toleration and the nature of phytoconstituents found in the extracts.

Moreover, the antimicrobial effect of *Citrus* essential oils was covered in the literature. Essential oils from leaves/twigs, small branches, wooden branches, and branch bark of *C. aurantium* were tested against *Agrobacterium tumefaciens*, *Dickeya solani* and *Erwinia amylovora* [48]. The authors reported an increase in inhibition zone diameters while increasing the amount of oil from 10 to 25 µL. In another study, the antibiofilm activity of *C. aurantium* essential oils was observed against *Stenotrophomonas maltophilia*, *Bacillus subtilis*, *Penicillium crustosum*, *P. expansum* and *P. citrinum* with inhibition zones ranging approximately from 8 to 18 mm [49]. Finally, essential oils obtained from *C. aurantium* flowers also reduced the growth of *Streptococcus mutans* and reduced the mRNA expression of its virulence genes [50].

### 3.3. Anti-Cancer and Cytotoxic Effect

Secondary metabolites existing in *C. aurantium* can be used in treating prostate and lung cancers [51]. Different studies supported the evidence of the anti-cancer properties of *C. aurantium*. Its polysaccharides exhibited a remarkable enhancement in the immune activity by promoting the production of interleukin 6 (IL-6) and tumor necrosis factor α (TNFα) in RAW264.7 cells [52]. They also promoted the production of inducible nitric oxide synthase (iNOS) and interleukin-1β (IL-1β) by stimulating their mRNA expression levels [52]. When treating RAW264.7 cells, *C. aurantium* polysaccharides greatly enhanced the phosphorylation of p65, p38, c-Jun N-terminal kinase (JNK), and the extracellular signal regulated kinase (ERK) [53]. The effect of ichanexic acid and isolimonic acid isolated from *C. aurantium* ethyl acetate extract on the proliferation and apoptosis of HT-29 colon cancer cells was also investigated. The obtained results demonstrated that these compounds play a potential role in halting the cell cycle by increasing the cell counts in the G2/M stage [54].

Moreover, two major compounds, 5-hydroxy-6,7,3′,4′-tetramethoxyflavone (HTF) and limonexic acid (LA), isolated from *C. aurantium* flowers displayed inhibitory effects on SMCC-7721 cell lines at a concentration ranging between 12.5 and 200 μg/ml, and on B16 cell lines at a concentration between 6 to 50 μg/ml [55]. Histopathological examination proved that the uptake of *C. aurantium* peel extract greatly decreased fibrosis in cholestatic liver fibrosis-induced mice [56]. Biochemical analysis demonstrated that *C. aurantium* peel extract displayed anti-inflammatory and anti-apoptotic activities in these mice by decreasing aspartate transaminase, alanine transaminase, total bilirubin, gamma-glutamyl transferase, and thiobarbituric acid reactive substances concentrations.

### 3.4. Anti-Diabetic Effect

The effectiveness of bitter orange extracts on liver antioxidant defense in diabetic mice was reported. Blood glucose level greatly dropped in experimental diabetic mice treated with bitter orange extracts as compared to untreated diabetic mice [57]. Glutathione peroxidase, malondialdehyde, and nitric oxide activities were significantly reduced, whereas superoxide dismutase activities were increased in the liver of diabetic mice. Bitter orange extract was not only effective in enhancing the liver antioxidant activity but also in reducing liver damages as revealed by histological analysis in experimental diabetic mice in comparison with untreated diabetic mice [57]. *C. aurantium* flavonoids also play a role in modulated insulin signaling cascade by preventing the phosphorylation of GSK3β and the activation of Akt in 3T3-L1 cells [58].

In vivo hypolipidemic and hypoglycemic impacts of neohesperidin (NHP) isolated from *C. aurantium* were inspected. Fasting glucose, glycosylated serum protein, serum glucose, total cholesterol, triglyceride, and leptin levels were considerably reduced in KK-Ay diabetic mice treated with NHP as compared to C57BL/6 mice used as a normal control [59]. The authors concluded that NHP inhibited the accumulation of lipids in the liver of KK-Ay diabetic mice by inhibiting fatty acid synthase (FAS), and stearoyl-CoA desaturase 1 (SCD-1) gene expression, and enhancing the gene expression of acyl-CoA oxidase (ACOX).

### 3.5. Anti-Obesity Effect

Because of its role in controlling thermogenesis and adipogenesis, *C. aurantium* can be regarded as a possible anti-obesity agent [51]. A further study delved into the underlying mechanism of the anti-obesity properties of the two major constituents in *C. aurantium*: naringin and neohesperidin [60]. Feeding high fat diet-induced obese C57BL/6 mice with *C. aurantium* extract for eight weeks resulted in a considerable decrease in mice adipose tissue, body weight, and total serum cholesterol. In vitro studies confirmed these results, since a reduction in lipid droplets was noted in 3T3-L1 adipocytes treated with *C. aurantium*. A greater differentiation and an increase in PPARγ coactivator 1 alpha and uncoupling of protein 1 thermogenic factors were observed in primary cultured brown adipocytes treated with *C. aurantium*. The authors also observed that the suppression of the AMP-activated protein kinase alpha (AMPKα) inhibited the effects of *C. aurantium* in adipocytes. These results propose that the action of *C. aurantium* as an anti-adipogenic and a thermogenic agent relies on the AMPKα pathway [60].

*C. aurantium* extracts containing *p*-synephrine obtained from the fruit peels were used in combination with other compounds such as caffeine to promote weight loss. This combination proved to be effective in promoting weight loss by increasing thermogenesis and lipolysis in both animals and humans. However, few studies were done to test whether *C. aurantium* extracts alone can promote weight loss [61]. The protoalkaloid *p*-synephrine and bitter orange extracts are safe to be used at defined doses in food industries and dietary supplements to promote weight loss due to their role as appetite suppressants [62,63]. Lately, chloroform extracts from *C. aurantium* flowers inhibited 3T3-L1 cell differentiation and subsequently suppressed lipid accumulation, thus attenuating metabolic diseases in high fat diet mice [64].

### 3.6. Anxiolytic Effect

Many studies have highlighted the anxiolytic effects of *C. aurantium*. The effect of *C. aurantium* essential oils on anxiety associated with bone marrow aspiration procedure done on patients with chronic myeloid leukemia (CML) was studied. Inhalation of *C. aurantium* essential oils caused a reduction in the scores of State-Trait Anxiety Inventory (STAI) psychometric scale and changes in physiological measurements concluding its anxiolytic effect [65]. In another study, *C. aurantium* essential oils were tested in patients participating in crack cocaine withdrawal. The Analog Smoke Scale (HAS) and the Trait-State Anxiety Inventory (IDATE) were used to assess the psychological measures [66]. Results showed that patients who nebulize *C. aurantium* essential oils were able to maintain controlled anxiety levels during the Simulated Public Speaking (SPS) method used to evoke anxiety as compared to the control group [66].

## 4. Extraction Methods of Active Constituents from *C. aurantium*

Whether derived from plant parts or from their waste, extraction of the bioactive compounds contributes largely to the valorization of the primary material, and to reducing the negative environmental impacts related to the agro-industrial activity. As clearly stated in this review, these metabolites can be used in several commercial sectors such as food, beverage, cosmetic, pharmaceutical, and other industries. Thus, their recovery can be considered very profitable. A broad range of extraction methods exist. Nonetheless, no single method can be viewed as a standard one for the extraction of bioactive compounds from their original matrices [67]. Extraction yield is strongly affected by the experimental conditions. In the next section, several conventional and non-conventional extraction methods applied on *C. aurantium* plant parts will be discussed.

### 4.1. Hydrodistillation

Hydrodistillation is a conventional extraction method that is widely used to extract bioactive compounds and essential oils from plant materials. Three different methods of hydrodistillation exist, including water distillation, direct steam distillation, and water and steam distillation. Upon boiling, water and steam play a role in freeing the bioactive substances from plant materials [68]. Four main parameters affect the yield of essential oils produced by hydrodistillation: the nature of the plant material, the operating pressure, and the distillation time and temperature [69]. The hydrodistillation of aromatic plants results in the production of essential oils and hydrosol co-products. Hydrosols are normally used as flavoring agents in pastries and beverages in the Middle East and Mediterranean basin [15]. Essentials oils obtained by hydrodistillation contain highly valuable volatile components, whereas hydrosols contain condensed water and a limited amount of dissolved essential oils. The yield of orange blossom hydrosol, also known as orange blossom water, obtained from *C. aurantium* through distillation is 99.9%. Neroli essential oil constitutes only 0.1% of the distillation products. All parameters, including pressure, temperature, and distillation time must be controlled during the industrial extraction process. A distillation trap was used in the traditional extraction approach [69].

Hydrodistillation is used to extract essential oils and bioactive compounds from *C. aurantium* flowers [70,71,72,73,74], peels [12,40,75], and leaves [28].

### 4.2. Solvent Extraction

During this process, the solvent permeates into the solid matrix to dissolve the solute. Solvent extraction efficiency is affected by several properties including the type of the extraction solvent, the solvent/solid ratio, the particle size, and the extraction duration and temperature [76]. It is a technique characterized by its simplicity and low energy demand. Its major drawbacks are long maceration time and organic solvent consumption.

Aqueous hydrotropic solutions were used to extract limonoid aglycones from *C. aurantium* seeds [23]. Sodium cumene sulphonate (Na-CuS) and sodium salicylate (Na-Sal) were used in this study. Ethanol, methanol, and hot water were used to extract phenolic compounds from all *C. aurantium* parts [11,13,17,18,21,22,24,29]. Active constituents from *C. aurantium* fruits and juice were also extracted using 80% acetone solvent [77] and ether-pentane (1:1) [12] respectively.

### 4.3. Soxhlet Extraction

In this method, a holder (thimble) containing the dry sample is placed in a distillation flask filled with the solvent of interest. Solvent vapor passes through the material placed into the thimble and is liquefied in the condenser. A siphon will then aspirate the solution and unload it into the distillation flask when the flow level is reached; separation of solvent and solute takes place. The steps are repeated until complete extraction is attained [78,79]. Soxhlet extraction is usually associated with high extraction efficiency that requires less time and solvent consumption than conventional extraction techniques.

Several studies adopted the soxhlet apparatus as an extraction method. It was used to extract active compounds from *C. aurantium* seeds with hexane solvent [54], crude limonoids and hesperidin using acetone and petroleum ether, respectively [20]. It was also used to extract fatty acids from *C. aurantium* seed oil using n-hexane [19]. Likewise, soxhlet extraction was conducted to extract bioactive compounds from *C. aurantium* peels [38] and seeds powder using water, hexane, methanol, acetone, and chloroform [80]. Petroleum ether, chloroform and ethanol solvents were used to extract *C. aurantium* leaves [47] and ethanol solvent from blossoms [16].

### 4.4. Ultrasound-Assisted Extraction

Ultrasound-assisted extraction (UAE) is a non-conventional green extraction method that is used to extract active constituents such as polyphenols from plant materials. It consists of ultrasonic waves that trigger cavitation. This phenomenon provokes alteration and collapse of the cell walls, and an increase in the interaction between any solvent and the compounds released from the treated sample. UAE has many advantages over other methods, such as the reduction of organic solvents consumption, reduction in extraction time, and increase in the extract yield [81]. It is also characterized by its high reproducibility and the ease of handling [82], and it is thought to enhance the biological properties of extracts [83].

Ultrasound water bath apparatus was used to extract volatile components from the flowers of four *Citrus* species including *C. aurantium* using n-pentane:diethylether solvent [84]. Phenolic compounds from *C. aurantium* blossoms were recovered using an ultrasonic apparatus, reaching 96 mg GAE/g DW and exhibiting excellent radical scavenging activity [85]. Alternatively, the volatile components present in *C. aurantium* flowers were extracted using ultrasonic-assisted headspace solid phase microextraction (UA-HS-SPME) [74]. The yield of flavonoids from *C. aurantium* flowers extracted using UAE was 1.8% [86]. Extraction of polysaccharides from *C. aurantium* flowers under optimal conditions yielded 3.9% to 4.3% using distilled water as a solvent [52].

Hydroalcoholic extracts (methanol and ethanol extracts) were also isolated from several varieties of *Citrus* leaves including *C. aurantium* leaves using UAE. The total amount of flavonoids and phenols was greater in ethanolic than in methanolic extracts, and all the extracts showed antioxidant activity and a moderate toxicity against the brine shrimp [87]. Phenolic compounds were recovered from *C. aurantium* peels using UAE, with 50% aqueous ethanol resulting in the highest yield [88]. In regard to the pectin yield, it was greatly increased under optimal conditions (ultrasound power 150 W, pH 1.5, during 10 min), reaching 28% of peels extract [42].

The ultrasound-assisted aqueous two-phase extraction (UA-ATPE) method was used to extract naringin, synephrine, and neohesperidin from the fruitlets of *C. aurantium* [89]. UA-ATPE is an alternative method that combines the ultrasound assisted extraction with the aqueous two-phase extraction such as ethanol/salt. It is an effective technique that integrates the two-phase separation and the field enhanced effect. Additionally, it achieves the extraction and the purification process in a single step. The amount of neohesperidin, synephrine, and naringin obtained were 89, 11, and 7 mg/g of fruitlet powder, respectively [89]. Authors reported that this method has many advantages over conventional extraction methods including shortening of the extraction time and increasing the yield and the purity of the obtained compounds. UAE is regarded as a technique that is suitable to extract active compounds while maintaining a reduced environmental impact [90].

### 4.5. Microwave-Assisted Extraction

Microwave-assisted extraction (MAE) is among the diverse thermal technologies, that is preferred over conventional techniques thanks to a more effective heating, reduced time and cost, and fast energy transfer [91]. It is a relatively novel extraction technique that is used to extract bioactive compounds from agro-industrial wastes including polyphenols, pectin, and others [92]. In this approach, the mixture of plant material and solvent is heated in a microwave to accelerate the extraction process [93]. MAE followed by the headspace solid phase apparatus is also used to extract volatile oils from *C. aurantium* leaves [27].

Pectin was extracted from *C. aurantium* peels using citric acid aqueous solution using MAE [43]. Microwave steam distillation (MSD) showed better extraction efficiency of essential oils from *C. aurantium* peels than the conventional steam distillation. Eighteen essential oils were detected using MSD versus only seven detected using conventional steam distillation [75]. In addition, ethanolic extraction by the MAE method produced a greater amount of flavonoid from *C. aurantium* peels (102 mg/g DW) as compared to aqueous extraction by the maceration method (52 mg/g DW) [94]. The impact of several microwave assisted extractions—microwave-assisted hydro-distillation (MAHD), solvent microwave extraction (SLME), and solvent-free microwave extraction (SFME)—on the fluctuations of essential oils composition from *C. aurantium* blossoms was studied [95]. The major compounds identified using the three methods were linalool, linalool acetate, geranyl acetate, farnesol, and nerolidol. Patented in 2004, SFME is an environmentally friendly method that can be applied to extract essential oils from plant materials [96]. It is known to release less carbon dioxide in the atmosphere than the traditional methods [97,98].

### 4.6. Supercritical Fluid Extraction

Supercritical fluid extraction (SFE) is an innovative green technology, and an environmentally friendly method applied to recover active constituents from plant materials. The extraction is based on the use of a supercritical fluid, mostly carbon dioxide since it is inexpensive and recognized as a safe solvent that has several physiochemical properties [99]. Carbon dioxide can be associated with a co-solvent. The method is characterized by an increase in the selectivity toward some compounds (modified by the temperature and the pressure), and a decrease in the consumption of organic solvents [100]. *C. aurantium* peels were subjected to supercritical carbon dioxide method, with ethanol as a co-solvent, to recover the essential oils [101], or phenols, fatty acid esters, coumarins and terpenes derivatives [102]. Diethyl-ether was selected as a co-solvent in another study to obtain enriched bioactive compounds as compared to simple pressing from *C. aurantium* peels [103].

The following table (Table 1) summarizes the studies reporting the extraction methods applied on *C. aurantium* plant parts, along with the active constituents recovered.

## 5. Conclusions

A plethora of scientific investigations has undoubtedly confirmed the exceptional benefits offered by *C. aurantium* as compared to other *Citrus* species. Several comparative studies proved that *Bigarade* leaves, juice, or flowers display the highest levels of TPC, TFC, or antiradical activity. This is undeniably reflected by a particular collection of bioactive constituents that include phenols, flavonoids, alkaloids, vitamin C, essential oils and others. Beneficial effects of these secondary metabolites are attributed to their advantageous biological and medicinal effects including antioxidant, antimicrobial, anti-cancer, anti-diabetic, anti-obesity, and anxiolytic effects, along with their uses in cosmetic and functional food industries. Although most of the bioactive compounds are found throughout the entire *C. aurantium* plant, a separate screening reveals a different identity for every plant part in terms of phytochemical composition and percentages. Interestingly, some of *C. aurantium* processing byproducts, such as the peels remaining after juice extraction, represent considerable sources of remarkable biomolecules that can have further uses. Valorization of the residual biomass, considered as waste, is primordial for the emerging bioeconomy. Last but not least, extraction methods existing in the literature, used to recover the bioactive compounds from *C. aurantium*, were for the first time summarized in this review. Nonetheless, very few studies compared the efficiencies of these different extraction techniques to conclude with the added value of one technique over the others. Further analytical methodologies aiming at fractionation and purification of these active constituents are essential, in combination with advanced clinical studies in order to unravel their mechanisms of activity as well as to assess their safety through their interaction with biological models.

## Figures and Tables

**Table 1:** Extraction methods and active constituents of the different parts of *C. aurantium* plant.

Plant Part	Extraction Method	Solvent	Active Constituents	References
**Juice**	Solvent extraction	Ether-pentane (1:1)	- Monoterpene hydrocarbons: limonene (92%), α-phellandrene (2%), and α-thujene (1%) - Oxygenated sesquiterpenes with caryophyllene oxide (1.4%) - Phenolic acids (71%) containing *p*-coumaric and ferulic acids (18% and 19%, respectively) - Flavonoid (23%) with rutin (6%) as the most frequent component	[12]
	Solvent extraction	80% methanol	- Total phenolic content 295 ± 4 mg GAE/g of fresh juice - Flavonoid content 26 mg Eq Q/g of fresh juice	[13]
	Solvent extraction	80% methanol	- 86% of phenolic compounds	[11]
**Flowers**	UAE	n-pentane:diethylether	- Linalool was the most abundant volatile compound (81%)	[84]
	UAE	Ethanol	- Flavonoid yield 2%	[86]
	Solvent extraction	Ethanol, methanol, and hot water	- Gallic acid, caffeic acid, syringic acid, rutin, pyrogallol, naringin and quercetin	[17]
	HD	Cyclohexane	- Linalool, linalyl acetate, geraniol, α-terpineol, and neroli oils	[73]
	UA-HS-SPME	Distilled water	- Fifty-four volatile compounds were identified	[74]
	HD	n-hexane	- Orange blossom water 99.9% - Neroli essential oil only 0.1% of the distillation product	[15]
	MAHD, SLME, SFME	Water	- Linalool, linalool acetate, geranyl acetate, farnesol, and nerolidol	[95]
	UAE	Ethanol	- TPC yield 96 mg GAE/g DW	[85]
	UAE	Distilled water and citric acid	- Pectin yield: 28% (of which 65% was of galacturonic acid)	[42]
	UAE	Distilled water	- Polysaccharide’s yield ranging between 3.9% and 4.3%	[52]
	Soxhlet extraction	Ethanol	- Highest TPC and TFC were recorded in *C. aurantium* flowers’ ethanolic extract with a value of 81 ± 3 mg GAE/g and 20 ± 3 mg QE/g of extract, respectively - Oxygenated monoterpenes (25%) mainly linalool, terpineol, hotrienol and nerol - Aliphatic hydrocarbons (18%) mainly tetrapentacontane followed by dotriacontan - Monoterpene hydrocarbons (15%) mainly limonene, β-pinene, β-ocimene and β-myrcene - Esters (12%) mainly linalyl acetate and neryl acetate	[16]
	Solvent extraction	80% ethanol	- D-glucuronic acid, D-limonene, octadecenoic acid, daphnetin, hexadecanoic acid, linalool, pyrrolidinone, and phthalic acid (10%, 5.5%, 4%, 3.7%, 2%, 2%, 1.2%, and 1% respectively)	[18]
**Seeds**	Solvent extraction	Na-CuS and Na-Sal	- Limonin 0.6 mg/g DW using Na-CuS, and 0.5 mg/g DW using Na-Sal	[23]
	Solvent extraction	Pure methanol	- Flavonoids: epigallocatechin, naringin, hesperidin, neohesperidin, naphtorecinol, quercetin, resorcinol, apigenin, kaempherol, catechin, and rutin - Phenolic acids: vanillic acid, syringic acid, gallic acid, rosmarinic acid, *p*-coumaric acid, and trans-2-hydroxycinnamic acid	[21]
	Soxhlet extraction	Acetone and petroleum ether	- The most abundant flavonoids in *C. aurantium* seeds are hesperidin, neohesperidin, naringin, narirutin	[20]
	Soxhlet extraction	n-hexane	- Linoleic acid (50%) and oleic acid (30%), cerotic acid, stearic acid, arachidic acid, palmitic acid, and palmitoleic acid	[19]
	Solvent extraction	Methanol	- Hydroxybenzoic acids (vanillic acid), and hydroxycinnamic acids (caffeic acid, trans-ferulic acid, and *p*-coumaric acid)	[22]
**Leaves**	UAE	Ethanol and methanol	- More chemical components in ethanolic than in methanolic extract	[87]
	MAE followed by HS-SPME	Water	- Fifty-three compounds including limonene and linalool	[27]
	HD	*Not specified*	- Flavanoids, phytosterols, carbohydrates, saponins, volatile oil, tannins, terpenoids, and proteins. Essential oils including eucalyptol (43%), sabinene (17%), β-linlool (15%), α-terpineol (8%), α-pinene (1.3%), β-myrcene (1.2%), 4-terpineol (1.1%), β- pinene (1%), D-limonene (1%), O-cymene (1%)	[28]
	Solvent extraction	Methanol:water (80:20)	- Total phenols (44 mg GAE/g DW), flavonoids (3 mg QE/g DW), flavonols (1.5 mg QE/g DW), proantho-cyanidins (4.5 mg CE/g DW), hydrolyzable tannins (33 ± 2 mg TAE/g DW), polymerized phenols (8 mg TAE/g DW), and soluble phenols (3 mg GAE/g DW)	[29]
	Solvent extraction	Absolute methanol	- TPC 70 ± 2 mg GAE/g DW in aqueous extract, 8 mg GAE/g DW in methanolic extract - TFC 12 ± 2 mg QE/g DW in aqueous extract, 5 mg QE/g DW in methanolic extract	[24]
	Solvent extraction	Methanol:water (80:20)	- TPC 107 ± 2 mg GAE/g DW - Flavonoids 14 mg Eq Q/g DW	[13]
**Peels**	SFE CO_2_	CO_2_ with ethanol as co-solvent	- Water was the most abundant fraction - The subsequent fractions were rich in essential oils	[101]
	HD	*Not specified*	- Monoterpene hydrocarbons (72.5%), oxygenated monoterpenes (7%) with limonene as the major volatile component (66%)	[40]
	HD	Diethyl ether	- Limonene (90%), phenolic acids (74%) mainly *p*-coumaric (25%) and ferulic acids (24%), flavonoids (23%)	[12]
	SFE CO_2_	CO_2_ with diethyl ether	- Limonene was the most abundant compound (54%) - Essential oils: linalyl acetate, linalool, geranyl acetate sabinene, and isogeijerin (greater concentrations in *C. aurantium* than in *C. sinensis*)	[103]
	MAE	Citric acid aqueous solution	- Pectin yield: 29% (of which 71% was of galacturonic acid)	[43]
	Soxhlet and SD	Hexane	- D-limonene (94%), and β-myrcene (3%)	[38]
	SD	Water	- Seven essential oils: α-pinene, β-pinene, myrcene, limonene, δ-carene, cyclohexene, α-terpineol, and cyclotrisiloxane	[75]
	MSD	Water	- Eighteen essential oils: α-pinene, β-pinene, myrcene, limonene, cyclohexene, α-terpineol, cyclotrisiloxane, sabinene, octyl aldehyde, n-octanol, n-nonadecanoic, hexasiloxane, thyocynic acid, dimethoxybenzylidene, γ-gurjunene, δ-guaiene, n-decanal, β-fenchyl alcohol	[75]
	SFE CO_2_	CO_2_ with ethanol as co-solvent	- Phenols, fatty acid esters, coumarins, and terpenes derivatives	[102]
	MAE	70% aqueous ethanol	- Greater amount of flavonoid content (102 mg/g DW) as compared to aqueous extraction by maceration method (52 mg/g DW)	[94]
	UAE	- 50% aqueous ethanol - 96% aqueous ethanol - 100% water	- Highest TPC recovered using 50% aqueous ethanol - Naringin, neohesperidin, caffeic acid, chlorogenic acid, coumaric acid, diosmin, ellagic acid, hesperidin, morin	[88]
	Peel rinds squeezing	*n*-hexane	- Limonene (49%), linalool (32%), linalyl acetate (12%), myrcene (1.2%), geranial (1%), neral (0.5%), β-pinene (0.5%), γ-terpinene (0.4%), sabinene (0.3%), geranyl acetate (0.2%), and β-caryophyllene (0.1%)	[41]
	HD	*Not specified*	- The essential oil content in *C. aurantium* peels ranged between 0.1 and 1.7% - The major constituents were limonene (68%–95%), followed by 1, 8- cineole (trace—15%), camphor (0.2–6%), α-terpinene (1–2%), octanal (0.02–2%), and α-terpinyl acetate (1–1.7%)	[36]
**Fruit**	UA-ATPE	Ethanol	- Neohesperidin 89 mg, synephrine 11 mg, and naringin 7 mg per g of fruitlet powder	[89]
	Solvent extraction	80% acetone	- Fifty-eight phenolic compounds comprising flavonoid glycosides, polymethoxylated flavonoids, coumarins, and phenolic acids	[77]
